# Serum 25(OH)D3 level is associated with cognitive impairment in middle-aged and elderly patients with type 2 diabetes mellitus

**DOI:** 10.3389/fendo.2026.1803549

**Published:** 2026-04-17

**Authors:** Xin Wu, Xiaoyang Wei, Lei Zhang, XiaoFang Han

**Affiliations:** 1 Anhui Medical University, Hefei, Anhui, China; 2 Department of Endocrinology, Hefei Second People’s Hospital, Hefei, Anhui, China

**Keywords:** 25(OH)D3, cognitive function-related factors, cognitive impairment, middle-aged and elderly people, neurobiological association, type 2 diabetes mellitus

## Abstract

**Objectives:**

This study aimed to investigate the association between serum 25-hydroxyvitamin D3 (25(OH)D3) levels and cognitive impairment (CI) in middle-aged and elderly individuals with type 2 diabetes mellitus (T2DM). Additionally, we sought to evaluate the potential of serum 25(OH)D3 as a marker associated with CI and to identify relevant influencing factors.

**Methods:**

This cross-sectional study included 221 middle-aged and elderly patients with T2DM, aged 50–80 years. Participants were divided into a group without cognitive impairment (n = 131, 59.28%) and a group with cognitive impairment (n = 90, 40.72%) based on cognitive function status. Demographic characteristics, clinical indicators (e.g., age, disease duration, body composition, comorbidities), and laboratory test results (e.g., 25(OH)D3, fasting C-peptide, fasting insulin, blood lipids) were collected. Logistic regression analysis was used to examine the association between 25(OH)D3 and cognitive impairment. Analyses included univariate logistic regression, multivariate regression adjusted for three sets of confounders, subgroup analyses, interaction tests, and ROC curve analyses.

**Results:**

The two groups showed statistically significant differences in age, disease duration, fasting C-peptide, fasting insulin, cholesterol, serum 25(OH)D3 levels, and 25(OH)D3 quartile distribution (P < 0.05). Differences in other indicators were not statistically significant (P > 0.05). Logistic regression analysis revealed that higher serum 25(OH)D3 levels were significantly associated with a lower prevalence of cognitive impairment. This association remained robust after adjusting for multiple confounders (including gender, hypertension, cerebral infarction, and coronary heart disease) in Model 3 (OR = 0.92, 95% CI: 0.88–0.97, P < 0.001). In the 25(OH)D3 quartile subgroup analysis, compared with the Q1 group, the Q3 (OR = 0.35, P = 0.034) and Q4 (OR = 0.25, P = 0.004) groups showed significantly lower odds of cognitive impairment, with a significant trend (P = 0.003). Receiver operating characteristic (ROC) curve analysis revealed an area under the curve (AUC) of 0.65 (95% CI: 0.58–0.72) for 25(OH)D3 in identifying cognitive impairment. Interaction tests indicated that factors such as gender, hypertension, cerebral infarction, and fatty liver did not significantly modify the association between 25(OH)D3 and cognitive impairment (P for interaction > 0.05).

**Conclusions:**

This study found that 25(OH)D3 levels were negatively correlated with the prevalence of cognitive impairment in middle-aged and elderly individuals with T2DM. Higher 25(OH)D3 levels are associated with lower odds of cognitive impairment, and 25(OH)D3 may have potential as a marker for cognitive impairment in this population. These findings highlight 25(OH)D3 as a potential factor of interest for further research into the prevention and intervention of cognitive impairment in middle-aged and elderly patients with T2DM.

## Background

As the global population ages at an accelerated pace, type 2 diabetes mellitus (T2DM) and cognitive impairment have emerged as core challenges in managing the health of middle-aged and elderly populations. The latest data from the International Diabetes Federation (IDF) indicates that approximately 589 million adults aged 20–79 worldwide have diabetes, a figure projected to rise to 853 million by 2050. Among these, one in four individuals with diabetes is aged 65 or older (1). Notably, individuals with T2DM are at high risk of cognitive impairment, with epidemiological studies confirming a prevalence of 45% in this population (2). Compared with the general population, the risk of developing mild cognitive impairment is increased by 60%, while the risk of dementia is increased by 50% to 100% (3). Furthermore, the prevalence of these conditions increases significantly with age (4).

Cognitive impairment refers to the deterioration of one or more functions across multiple domains, including memory, attention, executive function, language, literacy, reasoning, calculation, and orientation (5). Among diabetic populations, individuals with concurrent cognitive impairment exhibit reduced capacity for self-management of blood glucose levels, heightened risk of complications such as diabetic nephropathy and retinopathy, and the formation of a vicious cycle of “diabetes-cognitive impairment.” This places a significant burden on patients’ families and the healthcare system. Therefore, identifying modifiable factors associated with cognitive impairment in middle-aged and elderly T2DM patients holds significant clinical and public health value for identifying individuals at risk of cognitive decline and improving disease prognosis. Vitamin D3 is a lipophilic steroid with endocrine, autocrine, and paracrine activities (6). Recent studies have shown a close association between vitamin D3 and central nervous system function as well as glucose metabolism (7, 8). As the primary and most stable metabolite of vitamin D3, 25(OH)D3 serves as a reliable biomarker for assessing individual vitamin D3 status, offering a potential avenue for further investigation for cognitive impairment in T2DM patients.

Although several studies have explored the association between vitamin D3 and cognitive impairment—for instance, Llewellyn et al. reported that low vitamin D3 levels (<10 ng/mL or 25 nmol/L) were associated with an increased risk of cognitive decline in 858 community-dwelling elderly individuals in Italy (8)—research focusing on the specific high-risk group of middle-aged and elderly patients with T2DM remains scarce. This study aims to provide scientific evidence for the early identification and potential targeted intervention (such as vitamin D3 supplementation) of cognitive impairment in middle-aged and elderly T2DM patients, while enriching theoretical research on “metabolic–neural” cross-regulation.

## Study design and methods

### Study population

**Figure 1:**
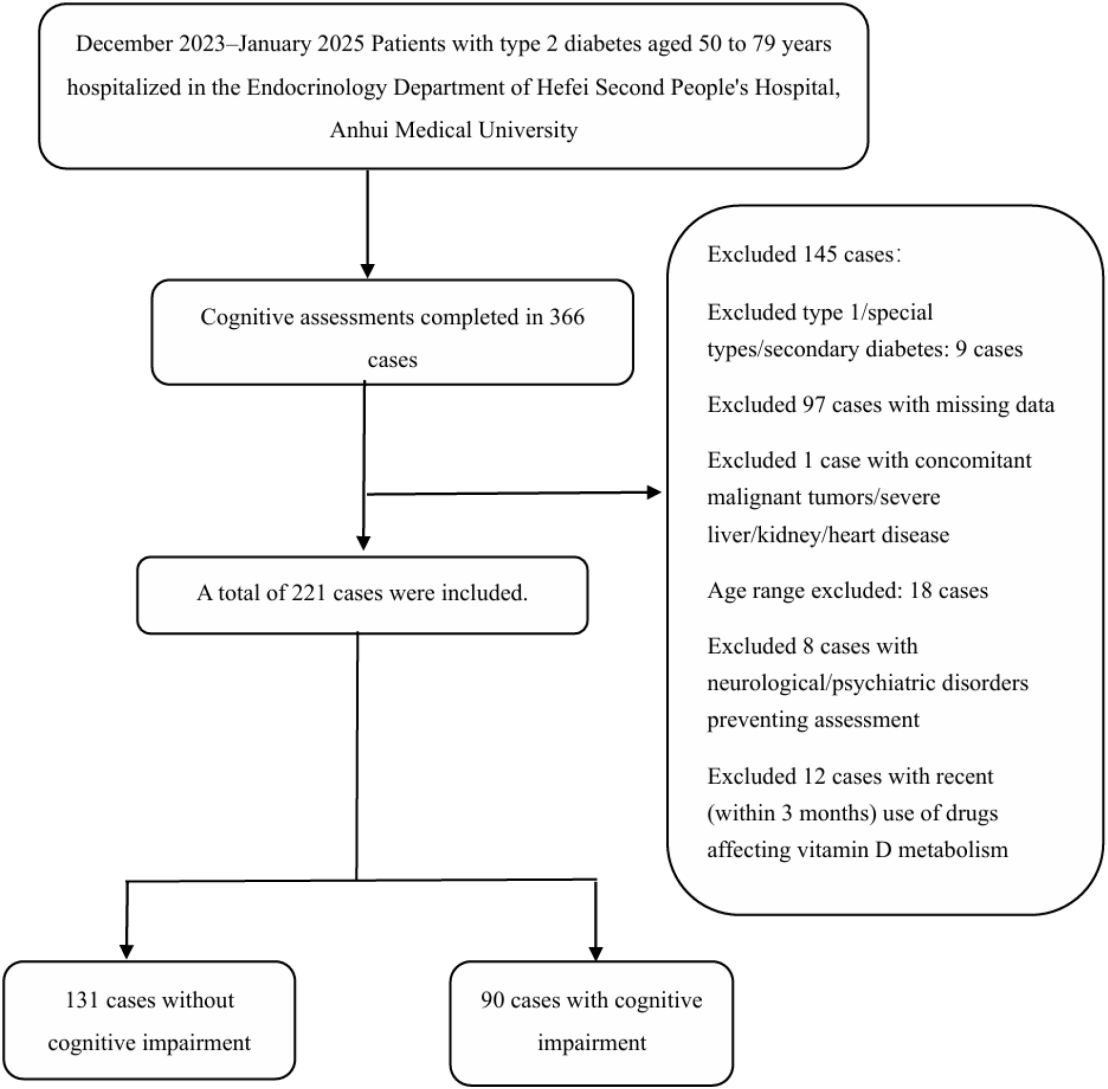
Flow diagram of participant enrollment and exclusion. Between December 2023 and January 2025, 366 inpatients with type 2 diabetes completed cognitive assessments. A total of 145 participants were excluded according to the study criteria, among which 97 cases were excluded due to missing 25(OH)D_3_ test data. Finally, 221 participants were included in the statistical analysis, consisting of 131 cases without cognitive impairment and 90 cases with cognitive impairment.

This cross-sectional study enrolled consecutive patients aged 50–80 years with T2DM who attended Hefei Second People’s Hospital from December 2023 to January 2025. Inclusion criteria: (1) Clinically diagnosed T2DM meeting the 1999 World Health Organization (WHO) diagnostic criteria; (2) Age between 50 and 80 years; (3) Being conscious and able to cooperate with cognitive function scale assessments, questionnaires, and laboratory indicator collection. Exclusion criteria: (1) Concurrent type 1 diabetes, special types of diabetes, or secondary diabetes; (2) Coexisting malignant tumors or severe liver, kidney, or heart disease; (3) History of neurological disorders such as Alzheimer’s disease, Parkinson’s disease, intracranial tumors, traumatic brain injury, or epilepsy; or presence of psychiatric conditions preventing assessment cooperation; (4) Use of medications potentially affecting vitamin D metabolism (including vitamin D3 supplements, glucocorticoids, or antiepileptic drugs) within the past 3 months. A total of 221 participants were ultimately included. The detailed process of participant enrollment and exclusion is illustrated in **Figure 1**.

### Methods

General and Clinical Data Collection: General demographic and clinical information for all participants was collected via structured questionnaires and electronic medical records, including gender, age (years), and educational attainment (illiterate/elementary school/junior high school and above). Physical measurements were performed using standardized protocols, including height (m), weight (kg), body mass index (BMI, kg/m²), waist circumference (cm), and abdominal circumference (cm).Clinical information included: T2DM duration (years), lifestyle (daily physical activity duration in hours, defined as cumulative time of moderate-to-vigorous intensity activity), and comorbidities (hypertension, cerebral infarction, coronary heart disease, fatty liver disease, diabetic nephropathy, diabetic retinopathy—all diagnosed according to the Chinese Guidelines for the Prevention and Treatment of Type 2 Diabetes (2022 Edition) and corresponding clinical diagnostic criteria). Laboratory parameters were collected from venous blood after 12-hour fasting and analyzed using a standardized testing system: 25(OH)D_3_ (ng/mL), fasting blood glucose (FBG, mmol/L), glycated hemoglobin (HbA1c), fasting insulin (FINS, uIU/mL), fasting C-peptide (F-CP, nmol/L), C-reactive protein (CRP, mg/L), serum albumin (ALB, g/L), creatinine (Cr, μmol/L), alanine aminotransferase (ALT, U/L), and aspartate aminotransferase (AST, U/L). Serum 25(OH)D3 levels were categorized into three clinically established groups according to the Endocrine Society clinical practice guidelines: deficiency (<20 ng/mL), insufficiency (20–29 ng/mL), and sufficiency (≥30 ng/mL).These indicators were used to assess glucose metabolism (FBG, HbA1c, FINS, F-CP), nutritional status (ALB), renal function (Cr), and hepatic function (ALT, AST), respectively. Serum 25(OH)D3 measurements were conducted from December 2023 to January 2025, and blood draw seasons were classified according to the standard meteorological division of the Northern Hemisphere (a universal criterion for clinical vitamin D research): winter (December 2023–February 2024 + January 2025), spring (March–May 2024), summer (June–August 2024), and autumn (September–November 2024). The seasonal distribution of blood draws among the 221 valid participants was as follows: winter (n=62, 28.05%), spring (n=55, 24.89%), summer (n=58, 26.24%), and autumn (n=46, 20.81%).

Cognitive function assessment: The MoCA was administered by a single professionally trained clinician. The cutoff values for cognitive impairment used in this study were established and validated in a large population-based study of elderly Chinese individuals by Jia’s team (9), which are widely adopted in Chinese clinical and research cognitive screening practices: ≤13 points for illiterate participants, ≤19 points for those with primary school education, and ≤24 points for those with junior high school education or above. This culturally and linguistically adapted Chinese version of MoCA has been verified to have favorable sensitivity (83.8% for all cognitive impairments, 80.5% for mild cognitive impairment) and specificity (82.5% for cognitively normal individuals) in elderly Chinese populations, thus ensuring its applicability to our cohort of middle-aged and elderly Chinese patients with T2DM. The clinician responsible for MoCA assessment received standardized professional training in neurocognitive scale operation and pre-study calibration per the Chinese MoCA operational guidelines. A strict standardized assessment protocol was followed throughout the study, and all scoring records were double-checked by the assessor to ensure the accuracy and consistency of the single-rater evaluation. Notably, the MoCA scale is the most widely used cognitive screening tool in clinical and research settings for middle-aged and elderly T2DM patients, specifically validated for detecting cognitive impairment in this high-risk population (2, 3). Its utility in identifying associations between modifiable risk factors (e.g., vitamin D status) and cognitive outcomes has been consistently supported by previous epidemiological studies (10, 12), confirming that it provides sufficient sensitivity and specificity for the core objective of this study—exploring the association between serum 25(OH)D3 levels and cognitive impairment.

### Statistical analysis

Statistical analysis was performed using SPSS software version 27.0. Prior to analysis, missing values were screened, with all variables exhibiting a missing rate <5%. Multiple imputation was used to handle missing data, ensuring the reliability of the analysis. Continuous variables are presented as mean ± standard deviation, and intergroup differences were assessed using the Mann-Whitney U test. Categorical variables were summarized as counts and percentages [n(%)], with intergroup comparisons performed using the chi-square test (χ² test). Spearman’s correlation analysis was employed to assess associations between variables. To investigate the association between 25(OH)D3 levels and cognitive impairment, multivariate logistic regression analysis was conducted, controlling for three models: Model 1: Unadjusted for confounders. Model 2: Adjusted for gender, age, and disease duration. Model 3: Adjusted for gender, hypertension, cerebral infarction, coronary heart disease, fatty liver, age, fat mass ratio (FMR), body mass index (BMI), diabetes duration, fasting C-peptide, glycated hemoglobin (HbA1c), alanine aminotransferase (ALT), aspartate aminotransferase (AST), triglycerides, total cholesterol, and blood draw season (winter/spring/summer/autumn as categorical variables).Results are reported as odds ratios (OR) and 95% confidence intervals (CI). Additionally, a trend analysis was conducted using the Cochran-Armitage test, with 25(OH)D3 quartiles as categorical independent variables and Q1 as the reference group, to examine the dose-response relationship. Subgroup analyses and interaction tests were conducted. Likelihood ratio tests assessed potential factors modifying the association between 25(OH)D3 and cognitive impairment (P for interaction). Receiver operating characteristic (ROC) curve analysis evaluated the ability of 25(OH)D3 to discriminate between individuals with and without cognitive impairment, calculating area under the curve (AUC), 95% CI, sensitivity, and specificity. Additionally, restricted cubic spline (RCS) modeling was employed to assess potential nonlinear associations between 25(OH)D3 and cognitive impairment risk. Finally, subgroup analyses were conducted for participants with different genders, ages, diabetes duration, hypertension, fatty liver disease, diabetic nephropathy, and diabetic retinopathy to explore the consistency and stability of the association between 25(OH)D3 and cognitive impairment across different populations. P < 0.05 was considered statistically significant.

## Results

### Participant characteristics

This study initially recruited 366 middle-aged and elderly patients aged 50–80 years diagnosed with T2DM who completed cognitive function assessments at Hefei Second People’s Hospital. After excluding cases with missing laboratory data and those who did not meet the inclusion criteria, 221 valid participants were finally enrolled in the statistical analysis. Based on the results of the Montreal Cognitive Assessment (MoCA) scale, participants were divided into two groups: the non-cognitive impairment group (n=131, 59.28%) and the cognitive impairment group (n=90, 40.72%). As shown in **Table 1**, the mean age was 62.34 ± 7.28 years in the non-cognitive impairment group and 67.44 ± 7.11 years in the cognitive impairment group, with a statistically significant age difference between groups (P < 0.001). In terms of gender distribution, the non-cognitive impairment group consisted of 67 males (51.15%) and 64 females (48.85%), whereas the cognitive impairment group included 38 males (42.22%) and 52 females (57.78%).There was no statistically significant difference in gender composition between groups (P = 0.192). Comparison of serum 25(OH)D3 levels revealed 24.32 ± 7.51 ng/mL in the non-cognitive impairment group versus 20.17 ± 7.35 ng/mL in the cognitive impairment group, with a significant intergroup difference (P < 0.001). Their quartile distributions also showed a statistically significant difference (P = 0.007). Furthermore, no statistically significant differences were observed between groups in the incidence of comorbidities including hypertension, cerebral infarction, coronary heart disease, fatty liver disease, diabetic nephropathy, and diabetic retinopathy (all P > 0.05). Overall baseline characteristic analysis indicated statistically significant differences between groups in age, T2DM duration, fasting C-peptide, fasting insulin, cholesterol, serum 25(OH)D3 levels, and quartile distribution (P < 0.05).

**Table 1:** Baseline characteristics of T2DM patients with or without cognitive impairment.

Variables	Total (n = 221)	Without (n = 131)	With (n = 90)	P
Age (years)	64.42 ± 7.62	62.34 ± 7.28	67.44 ± 7.11	<.001
Fat Mass (kg)	33.19 ± 6.62	33.08 ± 6.67	33.35 ± 6.59	0.768
Muscle Mass (kg)	64.11 ± 7.38	64.05 ± 7.90	64.20 ± 6.60	0.882
FMR	0.54 ± 0.22	0.55 ± 0.25	0.54 ± 0.16	0.733
WC (cm)	88.82 ± 11.25	89.07 ± 11.25	88.47 ± 11.31	0.699
HC (cm)	95.91 ± 10.16	95.76 ± 10.22	96.11 ± 10.12	0.802
PA (h)	0.99 ± 1.14	0.95 ± 1.04	1.05 ± 1.26	0.526
MNA-SF (score)	13.07 ± 1.46	13.05 ± 1.48	13.09 ± 1.44	0.859
BMI (kg/m²)	24.46 ± 3.30	24.51 ± 3.46	24.40 ± 3.05	0.809
Diabetes Duration (years)	9.82 ± 6.81	9.02 ± 6.26	10.79 ± 7.32	0.022
FCP (nmol/L)	1.80 ± 1.22	1.95 ± 1.30	1.63 ± 1.11	0.023
FINS (uIU/mL)	58.15 ± 84.70	68.35 ± 104.48	46.15 ± 50.53	0.021
HbA1c (%)	8.85 ± 2.05	8.92 ± 2.20	8.76 ± 1.82	0.593
FBG (mmol/L)	7.98 ± 3.05	8.08 ± 2.92	7.84 ± 3.24	0.575
CRP ( (mg/L)	9.06 ± 16.47	8.53 ± 12.35	9.96 ± 21.90	0.627
Cr (mu mol/L)	66.64 ± 24.79	67.68 ± 25.51	65.12 ± 23.76	0.452
ALB (g/L)	41.27 ± 3.73	41.54 ± 3.71	40.87 ± 3.74	0.19
ALT (U/L)	21.19 ± 14.37	22.61 ± 15.85	19.09 ± 11.62	0.075
AST (U/L)	20.49 ± 10.47	20.77 ± 11.15	20.06 ± 9.42	0.623
TG (mmol/L)	1.83 ± 2.04	1.75 ± 1.15	1.94 ± 2.90	0.492
TC (mmol/L)	4.39 ± 1.24	4.52 ± 1.22	4.24 ± 1.25	0.048
HDL-C (mmol/L)	1.30 ± 0.31	1.31 ± 0.32	1.30 ± 0.30	0.855
LDL-C (mmol/L)	2.48 ± 1.01	2.53 ± 1.01	2.42 ± 1.01	0.45
25 (OH)D3 (ng/ml)	22.63 ± 7.71	24.32 ± 7.51	20.17 ± 7.35	<.001
Gender (n,%)				0.192
Male	105 (47.51)	67 (51.15)	38 (42.22)	
female	116 (52.49)	64 (48.85)	52 (57.78)	
Educational Attainment (n,%)				0.031
Illiterate	26 (11.76)	16 (12.21)	10 (11.11)	
Primary School	47 (21.27)	20 (15.27)	27 (30.00)	
Junior High School and Above	148 (66.97)	95 (72.52)	53 (58.89)	
Hypertension (n,%)				0.112
no	80 (36.20)	53 (40.46)	27 (30.00)	
yes	141 (63.80)	78 (59.54)	63 (70.00)	
CIinf (n,%)				0.121
no	51 (23.08)	35 (26.72)	16 (17.78)	
yes	170 (76.92)	96 (73.28)	74 (82.22)	
CAD (n,%)				0.683
no	201 (90.95)	120 (91.60)	81 (90.00)	
yes	20 (9.05)	11 (8.40)	9 (10.00)	
NAFLD (n,%)				0.546
no	110 (49.77)	63 (48.09)	47 (52.22)	
yes	111 (50.23)	68 (51.91)	43 (47.78)	
DN (n,%)				0.703
no	174 (78.73)	102 (77.86)	72 (80.00)	
yes	47 (21.27)	29 (22.14)	18 (20.00)	
DR (n,%)				0.235
no	157 (71.04)	97 (74.05)	60 (66.67)	
yes	64 (28.96)	34 (25.95)	30 (33.33)	
25 (OH)D3 quantile (n,%)				0.007
Q1	49 (22.17)	21 (16.03)	28 (31.11)	
Q2	58 (26.24)	32 (24.43)	26 (28.89)	
Q3	51 (23.08)	31 (23.66)	20 (22.22)	
Q4	63 (28.51)	47 (35.88)	16 (17.78)	
25 (OH)D3 status, n (%)				0.005
Deficiency (<20 ng/mL)	75 (33.94)	35 (26.72)	40 (44.44)	
Insufficiency (20–29 ng/mL)	100 (45.25)	61 (46.56)	39 (43.33)	
Sufficiency (≥30 ng/mL)	46 (20.81)	35 (26.72)	11 (12.22)	

25 (OH)D3, 25-hydroxyvitamin D3; ALB, albumin; ALT, alanine transaminase; AST, aspartate aminotransferase; BMI, body mass index; CAD, coronary artery disease; CI, cognitive impairment; CIinf, cerebral infarction; Cr, creatinine; CRP, C-reactive protein; DN, diabetes nephropathy; DR, diabetes retinopathy; FBG, fasting blood glucose; FCP, fasting C peptide; FINS, fasting serum insulin; FMR, fat mass ratio; HDL-C, high-density lipoprotein cholesterol; HC, hip circumference; HbA1c, glycosylated hemoglobin; LDL-C, low-density lipoprotein cholesterol; MNA-SF, Mini Nutritional Assessment-Short Form; NAFLD, non-alcoholic fatty liver disease; PA, physical activity; TC, total cholesterol; TG, triglycerides; WC, waist circumference.

25 (OH)D3 status was classified based on the Endocrine Society clinical practice guidelines: deficiency (<20 ng/mL), insufficiency (20–29 ng/mL), sufficiency (≥30 ng/mL).

### Correlation analysis between cognitive function and various parameters

**Table 2** presents the univariate analysis of confounding variables and cognitive impairment. According to Spearman’s correlation analysis, cognitive impairment in middle-aged and elderly patients with T2DM was significantly associated with serum 25(OH)D3 levels and multiple clinical indicators. Specifically, serum 25(OH)D3 levels showed a significant negative correlation with cognitive impairment (r_s = -0.265, P < 0.001), indicating that lower 25(OH)D3 levels are associated with a higher risk of cognitive impairment. Additionally, cognitive impairment showed a significant positive correlation with age (r_s = 0.303, P < 0.001) and diabetes duration (r_s = 0.130, P = 0.022), indicating that older age and longer diabetes duration are associated with a more pronounced risk of cognitive impairment. Furthermore, cognitive impairment was significantly negatively correlated with fasting insulin (r_s = -0.131, P = 0.028), fasting C-peptide (r_s = -0.130, P = 0.023), and total cholesterol (r_s = -0.113, P = 0.048).

**Table 2:** Correlation analysis between cognitive impairment and other variables.

	r	P value
Age	0.303	< 0.001
Diabetes Duration	0.130	0.022
FCP	-0.130	0.023
HbA1c	0.010	0.865
FINS	-0.131	0.028
FBG	-0.001	0.985
ALT	-0.079	0.168
TC	-0.113	0.048
25(OH)D3	-0.265	< 0.001
BMI	-0.076	0.185
FMR	-0.019	0.732
Hypertension	0.077	0.179
CIinf	0.070	0.219
NAFLD	-0.060	0.296

### Association between cognitive impairment and serum 25(OH)D3: a logistic regression analysis

As shown in **Table 3**, the results of the multivariate logistic regression analysis revealed the strength of the association between serum 25(OH)D3 levels and cognitive impairment in middle-aged and elderly T2DM patients. Cognitive function status was used as the dependent variable, with 25(OH)D3 incorporated into the model as both a continuous variable and a categorical variable divided into quartiles (Q1-Q4, with Q1 as the reference group). In the unadjusted model (Model 1), elevated serum 25(OH)D3 levels were significantly associated with a reduced risk of cognitive impairment in middle-aged and elderly T2DM patients (OR = 0.93, 95% CI: 0.89-0.96, P <0.001). In Model 2, this association remained stable (OR = 0.93, 95% CI: 0.89-0.97, P <0.001). The Q4 group (highest quartile) showed a significantly reduced risk of cognitive impairment (OR = 0.28, 95% CI: 0.12-0.65, P = 0.003). After further adjusting for multiple confounding factors including hypertension, cerebral infarction, and coronary heart disease (Model 3), the Q2 group showed no significant difference compared to the Q1 group (OR = 0.56, 95% CI: 0.23–1.39, P = 0.213). The Q3 group showed a significantly lower risk of cognitive impairment compared to the Q1 group (OR = 0.35, 95% CI: 0.14–0.92, P = 0.034); the Q4 group exhibited an even more pronounced reduction in cognitive impairment risk (OR = 0.25, 95% CI: 0.10–0.64, P = 0.004). For each 1 ng/mL increase in serum 25(OH)D3 levels, the risk of cognitive impairment was reduced by 8%, and this association remained statistically significant (OR = 0.92, 95% CI: 0.88-0.97, P <0.001). This indicates that the negative correlation between 25(OH)D3 levels and cognitive impairment risk is independent of the aforementioned confounding factors. Furthermore, trend analysis revealed a significant decreasing trend in cognitive impairment risk with increasing 25(OH)D3 levels even after controlling for confounders in Model 3 (trend test P = 0.003). These findings demonstrate that 25(OH)D3 levels are significantly associated with a decreased risk of cognitive impairment, suggesting a potential association with cognitive function in this population.

**Table 3:** Logistic regression analysis of the association between cognitive impairment and 25(OH)D3.

Variables	Model 1		Model 2		Model 3	
	OR (95%CI)	*P*	OR (95%CI)	*P*	OR (95%CI)	*P*
25(OH)D3	0.93 (0.89-0.96)	**<.001**	0.93 (0.89-0.97)	**<.001**	0.92 (0.88-0.97)	**<.001**
25(OH)D3 quantile						
Q1	1.00 (Reference)		1.00 (Reference)		1.00 (Reference)	
Q2	0.61 (0.28-1.31)	0.205	0.60 (0.26-1.37)	0.226	0.56 (0.23-1.39)	0.213
Q3	0.48 (0.22-1.07)	0.074	0.42 (0.18-1.01)	0.052	0.35 (0.14-0.92)	**0.034**
Q4	0.26 (0.11-0.57)	**<.001**	0.28 (0.12-0.65)	**0.003**	0.25 (0.10-0.64)	**0.004**
P for trend	**<.001**		**0.002**		**0.003**	

Bold values indicate statistically significant results (P < 0.05).

### Nonlinear association between 25(OH)D3 and cognitive impairment

**Figure 2:**
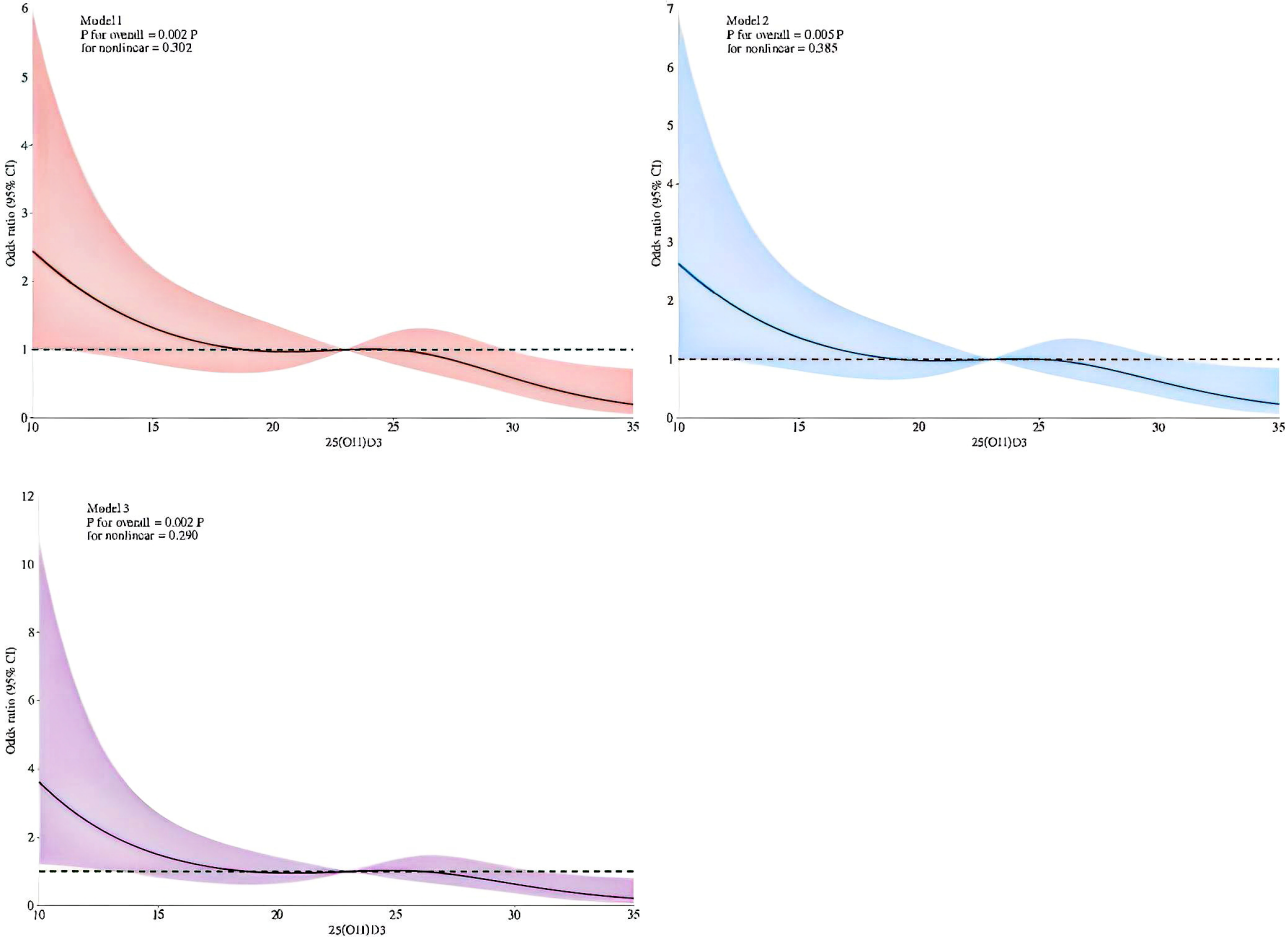
RCS analysis results.

Three restricted cubic spline (RCS) models were constructed to examine the relationship between serum 25(OH)D3 levels and cognitive impairment, controlling for three models (**Figure 2**). By controlling for four nodes, both serum 25(OH)D3 levels and the risk of cognitive impairment in middle-aged and elderly T2DM patients showed a significant linear association (P for nonlinear > 0.05). Across different models, this linear decreasing trend remained stable, with OR values uniformly decreasing as 25(OH)D3 levels increased, showing no signs of a threshold effect. In summary, the association between serum 25(OH)D3 and cognitive impairment risk is predominantly linear. For every 1 ng/mL increase in 25(OH)D3 levels, the risk of cognitive impairment steadily decreases, and this association remains robust across different adjusted models.

### Subgroup analysis and ROC analysis results

**Figure 3:**
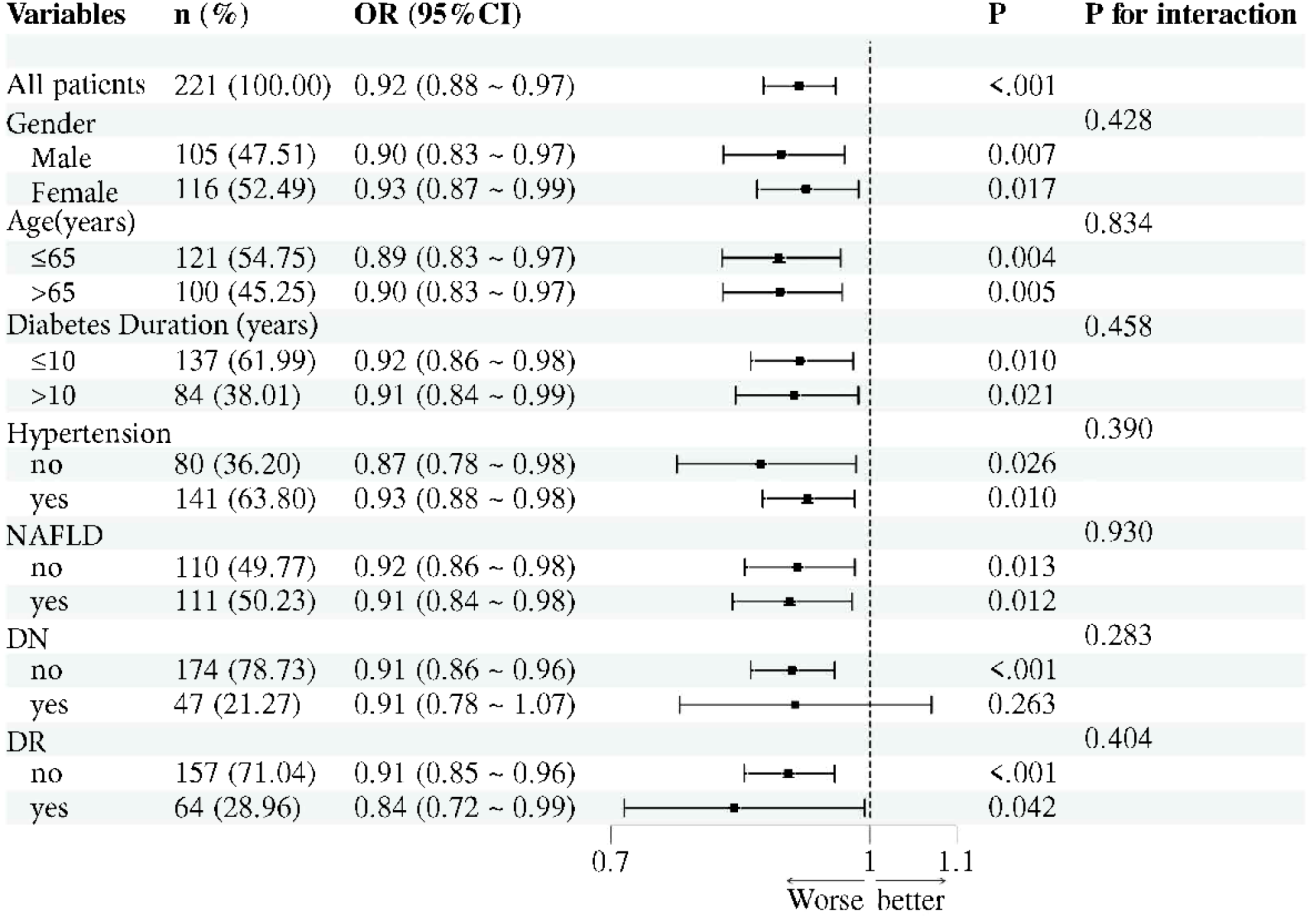
Forest plot analysis results.

Exploration of Potential Modifying Effects on the Association Between Serum 25(OH)D3 and Cognitive Impairment in Middle-Aged and Elderly T2DM Patients Across Different Subgroups (**Figure 3**). Analyses were conducted for participants varying in age, gender, diabetes duration, hypertension, fatty liver disease, diabetic nephropathy, and diabetic retinopathy. In all subgroups, interaction P-values were > 0.05. indicating that the association between 25(OH)D3 and cognitive impairment remains consistent and stable across different populations. The aforementioned factors did not significantly modify the association between 25(OH)D3 and cognitive impairment.

**Figure 4:**
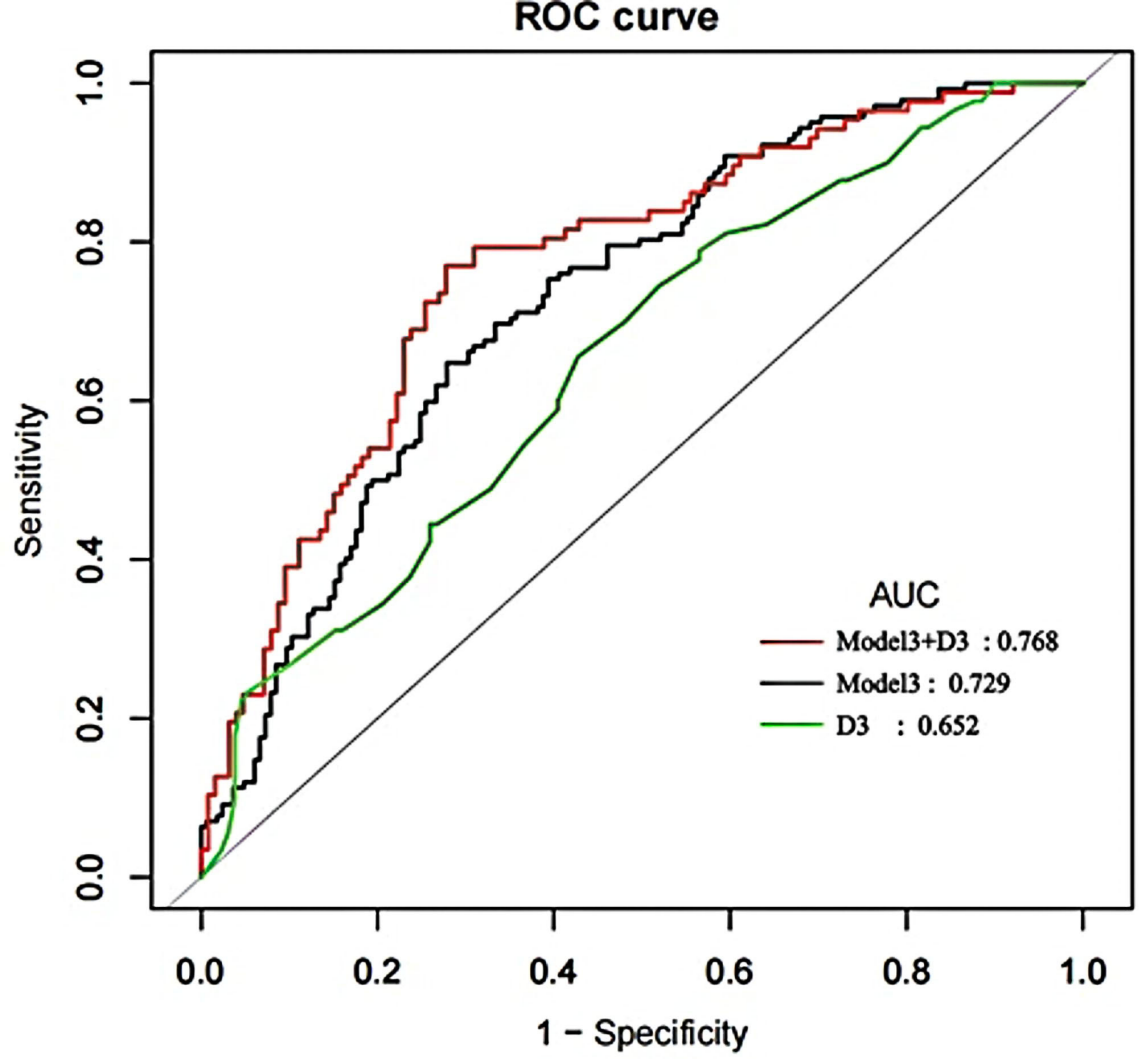
ROC curve analysis.

Further analysis using ROC curves assessed the ability of serum 25(OH)D3 to discriminate between individuals with and without cognitive impairment (**Figure 4**; **Table 4**): Model 3, adjusted for relevant variables, showed improved AUC to 0.7288 (95% CI: 0.6731–0.7846), with specificity 0.7212, sensitivity 0.6479, and accuracy 0.6873; Model 3 combined with 25(OH)D3 (Model 3+D3) demonstrated optimal discriminative performance, achieving an AUC of 0.7679 (95% CI: 0.7036–0.8323), specificity of 0.7222, sensitivity 0.7701, accuracy 0.7418. Additionally, positive-LR (2.7724), diagnosis-OR (8.71), and negative-pv (0.8198) were superior to those of individual indicators, suggesting that the combined model may have greater utility in identifying individuals with cognitive impairment.

**Table 4:** ROC analysis results.

Test	ROC area(AUC)	95%CI low	95%CI upp	Specificity	Sensitivity	Accuracy	Positive-LR	Negative-LR	Diagnose-OR	N-for-diagnose	Postive-pv	Negative-pv
Model3	0.7288	0.6731	0.7846	0.7212	0.6479	0.6873	2.3239	0.4882	4.76	2.7093	0.6667	0.7041
D3	0.6516	0.5789	0.7242	0.5725	0.6556	0.6063	1.5335	0.6016	2.549	4.3845	0.513	0.7075
Model3+D3	0.7679	0.7036	0.8323	0.7222	0.7701	0.7418	2.7724	0.3183	8.71	2.0311	0.6569	0.8198

### Sensitivity analysis

To assess the potential impact of unmeasured confounders on the study results, we employed E-value sensitivity analysis. Based on the association result between the highest quartile (Q4, n=63) of 25(OH)D3 and cognitive impairment in Model 3 (OR = 0.25, 95% CI: 0.10-0.64), the point estimate E-value was calculated as 7.46, with the upper bound of the 95% confidence interval corresponding to an E-value of 2.5.This indicates that an unmeasured confounder with an association strength at least 7.46 times greater than that observed for both 25(OH)D3 levels and cognitive impairment would be required to fully explain the observed protective effect. Even at the upper limit of the 95% confidence interval (OR = 0.59), an association strength at least 2.5 times greater would be necessary. The E-value results suggest that the study’s conclusion regarding the association between higher 25(OH)D3 levels and reduced risk of cognitive impairment is robust to unmeasured confounding factors.

## Discussion

The present study explored the association between serum 25(OH)D3 levels and cognitive impairment in middle-aged and elderly patients with T2DM (aged 50–80 years). Results showed that for every 1 ng/mL increase in serum 25(OH)D3 levels, the risk of cognitive impairment was significantly reduced (after adjusting for confounding factors) (OR = 0.92, 95% CI: 0.88–0.97, P < 0.001). High 25(OH)D3 levels (Q3 and Q4 groups) were associated with significantly lower odds of cognitive impairment compared with the lowest quartile (Q1) (Q3: OR = 0.35, P = 0.034; Q4: OR = 0.25, P = 0.004), demonstrating a clear dose–response relationship. 25(OH)D3 showed moderate discriminative ability for cognitive impairment (AUC = 0.65), and this association remained stable across different genders and comorbidity subgroups. For each 1 ng/mL increase in serum 25(OH)D3 levels, the risk of cognitive impairment decreased by 8% in middle-aged and elderly T2DM patients (adjusted OR = 0.92, 95% CI: 0.88–0.97, P < 0.001). Quartile subgroup analysis revealed that the Q3 and Q4 groups had 65% and 75% lower risks of cognitive impairment compared to the Q1 group (OR = 0.35, 0.25, P < 0.05 for both), with a trend test P = 0.003 confirming a clear dose-response relationship. Clinically, 33.94% of the participants had vitamin D deficiency and 45.25% had insufficiency, with cognitive impairment showing a significantly higher prevalence in the deficiency group (44.44%) than in the sufficiency group (12.22%), further supporting that maintaining serum 25(OH)D3 levels above 30 ng/mL may be a clinically meaningful target for further investigation in relation to cognitive impairment in middle-aged and elderly T2DM patients.

Numerous studies have explored the association between serum 25(OH)D3 levels and cognitive impairment, with most reporting that low serum 25(OH)D3 levels are associated with an increased risk of cognitive decline. A cross-sectional study by Annweiler et al. demonstrated that serum 25(OH)D3 levels in patients with mild cognitive impairment (MCI) were significantly lower than in cognitively healthy individuals (53.5 ± 21.8 nmol/L vs. 70.6 ± 34.2 nmol/L, P = 0.006). Furthermore, for every 1 nmol/L increase in 25(OH)D3 levels, the risk of developing MCI decreased by 4% (adjusted OR = 0.96, 95% CI: 0.93-0.98, P = 0.002). The risk of MCI in the lowest quartile group (10–40 nmol/L) was 25.46 times higher than in the highest quartile group (80–189 nmol/L) (P = 0.002), suggesting that 25(OH)D3 deficiency is associated with cognitive impairment at its earliest stage (MCI) (10). A cross-sectional study by Li et al. using NHANES (2011-2014) data further revealed a U-shaped relationship between serum 25(OH)D3 and biological aging (inflection point at 68.1 nmol/L). Individuals with low 25(OH)D3 (<50 nmol/L) combined with poor cognitive performance exhibited significantly increased biological aging risk (OR = 1.67, 95% CI: 1.22–2.27, P < 0.01), while normal 25(OH)D3 levels combined with normal cognitive function reduced the apparent age by 2.40 years, indicating a potential synergistic association with cognition-related aging processes (11). A cohort study by Littlejohns et al. (median follow-up 5–6 years) confirmed that individuals with severe serum 25(OH)D3 deficiency (<25 nmol/L) had a 2.25-fold increased risk of all-cause dementia (95% CI: 1.23–4.13, P<0.01) compared to those with adequate levels (≥50 nmol/L), and a 2.22-fold increased risk of Alzheimer’s disease (AD) (95% CI: 1.02–4.83, P < 0.05). Furthermore, individuals with insufficient 25(OH)D3 levels (25–<50 nmol/L) exhibited a 1.53-fold and 1.69-fold increased risk of all-cause dementia and AD, respectively (both P < 0.05), with a significant upward trend in risk observed at 25(OH)D3 levels <50 nmol/L (12). A 12-year follow-up study by Feart et al. further confirmed that individuals with 25(OH)D3 deficiency (<25 nmol/L) had a 2.85-fold higher risk of AD onset (95% CI: 1.36–5.97, P < 0.01) compared to those with adequate levels (>50 nmol/L), along with faster cognitive decline. This association remained stable after adjusting for confounding factors (13). A research team from Erasmus Medical Center in the Netherlands conducted the Rotterdam Prospective Population Cohort Study, enrolling 6,220 participants aged 55 years and older. They measured serum 25 -hydroxyvitamin D levels and followed them prospectively until 2015. After multivariable adjustment, low serum vitamin D levels were significantly associated with an increased risk of developing dementia (including Alzheimer’s disease), while no statistically significant association was found with the prevalence of dementia (14). These studies, spanning diverse populations, follow-up durations, and stages of cognitive impairment (from MCI to dementia), consistently indicate a strong association between serum 25(OH)D3 levels and cognitive function.

However, the relationship between serum 25(OH)D3 levels and cognitive impairment remains controversial, as several well-designed cohort studies have failed to detect such an association. An 18-year long-term follow-up study by Olsson et al. demonstrated that neither plasma 25(OH)D3 concentration, dietary vitamin D intake, nor vitamin D synthesis genetic risk score was significantly associated with the risk of all-cause dementia, Alzheimer’s disease, or cognitive impairment. After adjusting for confounding factors, the relative effect values remained close to 1.0 (hazard ratios [HR] range 0.86–1.04, all P > 0.05) (15). The Canadian Health and Aging Study, following 661 individuals aged 65 and older for 10 years, found no statistically significant association between plasma 25(OH)D3 levels and cognitive decline, all-cause dementia, or Alzheimer’s disease risk in the overall population. In the female subgroup, elevated 25(OH)D3 concentrations were even associated with a potential increase in dementia risk (Model 3 adjusted HR = 1.17, 95% CI: 1.01–1.36, P = 0.035) (16). Graf et al. reached similar conclusions in hospitalized elderly patients, finding no association between 25(OH)D3 deficiency (<25 nmol/L) and cognitive status (P>0.05) (17).The discrepant findings between these studies and our work stem from three key methodological and population-specific distinctions that merit critical discussion. First, study populations differed markedly: Olsson et al. (15) and the Canadian Health and Aging Study (16) focused on the general elderly without metabolic comorbidity stratification, while our research targeted middle-aged and elderly T2DM patients—a high-risk cohort for cognitive impairment (2, 3) with chronic insulin resistance and low-grade inflammation, which may amplify 25(OH)D3’s neuroprotective effects and strengthen its association with cognitive function. Second, study design and exposure assessment varied: Graf et al. (17) studied frail hospitalized older adults, in whom vitamin D deficiency may be secondary to severe illness or malnutrition (underadjusted confounders), whereas we enrolled stable ambulatory T2DM patients and excluded those with severe organ dysfunction, minimizing reverse causation bias. Additionally, these negative studies used alternative plasma 25(OH)D3 detection methods (e.g., radioimmunoassay), while we adopted a standardized chemiluminescent immunoassay for 25(OH)D3—a more validated approach for clinical research. Third, follow-up and outcome definitions differed: long-term cohort studies (e.g., Olsson et al. (15), 18 years) are prone to cumulative unmeasured confounders (e.g., vitamin D supplementation, lifestyle changes) that dilute baseline 25(OH)D3-cognitive impairment associations. In contrast, our cross-sectional design focused on current 25(OH)D3 status in metabolically vulnerable T2DM patients, where such associations are more detectable. These differences highlight the importance of population stratification in vitamin D-cognitive impairment research, and our findings confirm that 25(OH)D3’s neuroprotective effects may be more pronounced in metabolically high-risk subgroups like T2DM patients.

This study focuses on middle-aged and elderly patients with T2DM, a specific high-risk population exhibiting a cognitive impairment prevalence of 45% (2). This condition forms a vicious cycle of “diabetes-cognitive impairment” (4, 18), yet previous research has largely neglected exploratory investigations in this cohort. Furthermore, this study adds to the evidence that serum 25(OH)D3 is consistently associated with better cognitive outcomes in middle-aged and elderly T2DM patients, a population with complex metabolic disorders, and this negative association with cognitive impairment risk is strengthened with increasing 25(OH)D3 concentrations. This provides more stable evidence for further investigation of intervention strategies in this population.

The observed negative association between serum 25(OH)D_3_ levels and cognitive impairment risk may be explained by several potential biological mechanisms, which require further prospective verification. Previous studies have identified direct associations with protective effects on the central nervous system: Serum 25(OH)D_3_ is converted to 1,25(OH)_2_D_3_ in the kidneys, which can cross the blood-brain barrier. There, it binds to vitamin D receptors (VDR) widely distributed throughout the central nervous system, and this binding has been associated with regulation of neurotransmitter synthesis and release, improved synaptic plasticity, and neuronal survival (19, 20). Concurrently, 1,25(OH)_2_D_3_ inhibits activation of the nuclear factor-κB (NF-κB) pathway, which is associated with reduced release of inflammatory mediators such as tumor necrosis factor-α (TNF-α) and interleukin-6 (IL-6) (21–23). and the chronic low-grade inflammatory state present in T2DM patients is a key mechanism associated with cognitive impairment (24). Thus, serum 25(OH)D3 may be linked to reduced brain inflammatory damage through its anti-inflammatory effects, a potential biological mechanism that requires further validation. 25(OH)D3 is known to be associated with improved glucose metabolism and insulin sensitivity, which are in turn linked to better cognitive function: Insulin resistance is a core pathological feature of T2DM and has been associated with impaired cognitive function by affecting brain energy metabolism and promoting β-amyloid deposition (25). Vitamin D3 regulates pancreatic β-cell function and enhances insulin receptor sensitivity (26). In this study, fasting insulin and C-peptide levels showed significant negative correlations with cognitive impairment (r = -0.131, -0.130, P < 0.05), suggesting that 25(OH)D3 may be indirectly associated with better cognitive function via its correlation with improved insulin resistance, forming a potential regulatory link among vitamin D3, insulin sensitivity, and cognitive function. Antioxidant and blood-brain barrier protection, blocking injury pathways: Oxidative stress and vascular damage induced by hyperglycemia are major contributors to cognitive decline in T2DM patients (4, 27). Active vitamin D3 is associated with increased activity of antioxidant enzymes such as superoxide dismutase and glutathione peroxidase, scavenging reactive oxygen species and reducing oxidative stress-induced damage to neurons (28, 29). Concurrently, it has been associated with maintenance of blood–brain barrier integrity, preventing the entry of peripheral toxic substances into brain tissue (20), which may represent a potential biological pathway linked to the reduction of cognitive impairment risk, and this requires further experimental and clinical verification.

### Limitations of the study

This study has certain limitations. First, the cross-sectional design precludes the establishment of a causal relationship between serum 25(OH)D3 levels and cognitive impairment, and prospective cohort studies are thus needed to further validate these findings. Second, potential residual confounding remains inevitable: clinical data on depression, smoking status, and alcohol consumption—all established confounding factors for cognitive impairment in middle-aged and elderly populations—were not collected in this study. Although we excluded participants who had taken vitamin D3 supplements within the past three months and incorporated blood draw season as a categorical covariate in Model 3 of the multivariate logistic regression to adjust for seasonal variations in sunlight exposure, individual differences in outdoor activity duration, sun protection behaviors, and dietary vitamin D intake among participants may still independently affect serum 25(OH)D3 concentrations, leading to uneliminated residual seasonal confounding. Additionally, cognitive function was only assessed via a single MoCA screening without follow-up evaluations, which makes it difficult to comprehensively capture the dynamic characteristics of cognitive impairment. Finally, despite being sufficient for this cross-sectional study, the relatively small sample size may compromise the statistical power of subgroup analyses. Future research should conduct prospective cohort studies or randomized controlled trials to clarify whether vitamin D3 supplementation can reduce the incidence of cognitive impairment or slow its progression in patients with T2DM.

## Conclusions

In middle-aged and elderly patients with T2DM, low serum 25(OH)D3 levels are closely associated with an increased risk of cognitive impairment. Increased serum 25(OH)D3 levels are significantly associated with a lower risk of cognitive impairment in this cohort. This finding highlights serum 25(OH)D3 as a potential biomarker for future prospective research on the early identification and targeted intervention of cognitive impairment in middle-aged and elderly patients with T2DM, which holds significant clinical and public health implications.

## Data Availability

The original contributions presented in the study are included in the article/supplementary material. Further inquiries can be directed to the corresponding author.
